# Medical Residency in Gynecology and Obstetrics in Times of COVID-19: Recommendations of the National Specialized Commission on Medical Residency of FEBRASGO*

**DOI:** 10.1055/s-0040-1715147

**Published:** 2020-07

**Authors:** Gustavo Salata Romão, Lucas Schreiner, Claudia Lourdes Soares Laranjeiras, Zsuzsanna Ilona Katalin de Jarmy Di Bella, Raquel Autran Coelho, Maria da Conceição Ribeiro Simões, Mario Dias Correa Júnior, Milena Bastos Brito, Marcelo Luis Steiner, Alberto Trapani Junior, Ionara Diniz Evangelista Santos Barcelos, Alberto Carlos Moreno Zaconeta, Francisco José Candido dos Reis, Karen Cristina Abrão, Sheldon Rodrigo Botogoski, Giovana da Gama Fortunato, Lia Cruz Vaz da Costa Damasio, Marcos Felipe Silva de Sá, César Eduardo Fernandes, Agnaldo Lopes da Silva Filho

**Affiliations:** 1 Universidade de Ribeirão Preto, Ribeirão Preto, SP, Brazil; 2 Pontifícia Universidade Católica do Rio Grande do Sul, Porto Alegre, RS, Brazil; 3 Rede Mater Dei de Saúde, Belo Horizonte, MG, Brazil; 4 Escola Paulista de Medicina, Universidade Federal de São Paulo, São Paulo, SP, Brazil; 5 Universidade Federal do Ceará, Fortaleza, CE, Brazil; 6 Centro Universitário Aparício Carvalho, Porto Velho, RO, Brazil; 7 Faculdade de Medicina, Universidade Federal de Minas Gerais, Belo Horizonte, MG, Brazil; 8 Universidade Federal da Bahia, Salvador, BA, Brazil; 9 Faculdade de Medicina do ABC, Santo André, SP, Brazil; 10 Hospital Universitário, Universidade Federal de Santa Catarina, Florianópolis, SC, Brazil; 11 Universidade Estadual do Oeste do Paraná, Cascavel, PR, Brazil; 12 Faculdade de Medicina, Universidade de Brasília, Brasília, DF, Brazil; 13 Faculdade de Medicina de Ribeirão Preto, Universidade de São Paulo, Ribeirão Preto, SP, Brazil; 14 Escola de Ciências da Saúde, Universidade Anhembi Morumbi, São Paulo, SP, Brazil; 15 Pontifícia Universidade Católica do Paraná, Curitiba, PR, Brazil; 16 Universidade Federal do Paraná, Curitiba, PR, Brazil; 17 Universidade Federal de Mato Grosso, Cuiabá, MT, Brazil; 18 Universidade Federal do Piauí, Teresina, PI, Brazil

## Introduction

The COVID-19 pandemic (Coronavirus Disease 2019) has affected residencies training around the world. In the light of responses to the pandemic, many medical residencies are being forced to reorganize their rotations. These changes include a reduction in the operating room and outpatient care training, and the cancellation of some activities such as visits to the wards, simulation sessions, among others.^1^

According to recommendations of the American College of Surgeons (ACS),^1^ elective surgeries should be postponed during the pandemic. The major aspects that support this position include difficulties with mobilizing human resources, materials, and personal protective equipment (PPE) needed for these procedures, the high risk of contagion of the team and of postoperative complications in patients with COVID-19. Although it is not a consensus yet, the proposal was to consider postponing elective procedures and surgeries for up to 3 months, provided that no harm was caused to the clinical condition of the patients. More recently, the International Federation of Gynecology and Obstetrics (FIGO) has published a FIGO Statement about guidance for resuming elective surgeries at the current point of the COVID-19 health crisis.^2^ The American College of Obstetrics and Gynecology (ACOG) has also drawn up recommendations on how the program directors can support obstetrics and gynecology residents during the COVID-19 pandemic.^3^

In Brazil, on 8 May, 2020, the National Medical Residency Commission (CNRM, in the Portuguese acronym), linked to the Ministry of Education, released a Technical Note Draft with recommendations for medical residency programs during the pandemic.^4^ Such recommendations derived from guidelines established for medical residencies by CNRM Resolution number 2/2006 and the Competency Framework of each specialty for the redefinition of the rotations and distribution of the workload among activities. The guideline also includes the “Manifestation of the Federal Council of Medicine in relation to the COVID-19 pandemic”, published on 25 March 2020. Its recommendation is that Brazilian physicians remain in their jobs, because “in this position, they will be able to exercise their most relevant role as guardians of life.” The Federal Council of Medicine also deems necessary the provision of PPE for physicians and other health professionals by the government and health authorities, so these professionals can perform their work safely.

According to this document, medical residency programs should make its curriculum more flexible to suit the pandemic context. The legislation in force establishes the maximum workload of 60 hours per week per resident, with between 10 and 20% destined to classes and between 80 and 90% to practical activities, already including a maximum of 24 hours on duty. Besides, it provides the resident with a weekly day off. The National Medical Residency Commission recommends the development of the classes within the maximum allowed limit (20% of the total workload) of 12 hours per week. Face-to-face classes must be suspended and developed through virtual meetings using videoconferencing tools. Classes should address themes related to each medical specialty, and issues related to the pandemic. Such topics include the detection, management, and flow for the care of patients with COVID-19, its complications, and individual and collective protection strategies. Also, training for donning and removal of PPE, and orotracheal intubation techniques should be included in the syllabus of medical residencies.

The National Medical Residency Commission establishes that in clinical training, the relocation of medical residents during the pandemic should be based on an Incidence Coefficient (number of new cases/million inhabitants) and the classification by the epidemiological complexity level of the Municipality and Health Region of the medical residency program. Table 1 shows the main recommendations for the workload of the residents in fighting the pandemic according to the incidence of COVID-19 and the epidemiological complexity of the region.

**Table 1:** Recommendations of the National Medical Residency Commission for the relocation of medical residents in the fight against the pandemic

Level	Epidemiological complexity	Incidence coefficient	Workload in pandemic activities	% of the practical workload
**1**	Emergency	50% above national incidence	Up to 24h a week	Up to 50%
**2**	Attention	between 50% above and national incidence	Up to 20h a week	Up to 40%
**3**	Alert	Below national incidence	Up to 12h a week	Up to 25%

**Source:** Adapted from the Ministry of Education.^4^

For medical specialties related to the care of patients with COVID-19, the entire residency clinical workload can be used with care activities to combat the pandemic.

Clinical training in medical residency programs must be redistributed according to the clinical experience of the residents. The resident must practice under supervision, ensuring the maximum use of the training and preserving the patient safe. The reorganization of rotations must consider the availability and learning opportunities in clinical settings of the host institution and of institutions associated with the medical residency program, prioritizing the performance in hospital care and emergency rooms, and always respecting the maximum workload of the residents. After returning to normality, the replacement of the residency program activities that were not developed during the pandemic will be subject to analysis and to a subsequent decision by the National Medical Residency Commission.

Medical residents included in the risk group according to criteria established by the Ministry of Health, as well as pregnant women, must communicate their condition to the medical residency program supervisor and the respective Medical Residency Committee (COREME, in the Portuguese acronym) of the hospital. These individuals will be relocated according to the risk of infection or take a leave from clinical settings through sick leave, in which case the relevant medical report should be presented. In case of leave by suspicion or confirmation of COVID-19, regulations established by the Ministry of Health and health services in the states and municipalities must be followed and the resident must comply with the recommended isolation. The COREME must report the absence of the resident as “on sick leave” to the National Medical Residency Commission System (SISCNRM, in the Portuguese acronym) and notify the scholarship manager for the suspension of regular payment. Residents on sick leave must follow the procedure with the National Social Security Institute (Brazilian INSS). Other situations of leave of absence provided for in paragraphs 2, 3, 4 of Art. 4 of law number 12.514/2011 include paternity leave of 5 days and maternity leave of 120 days, extendable for another 60 days in agreement with the institution responsible for the medical residency program. The time required to complete the program must be extended by a period equivalent to the duration of the leave period of the resident.

In situations when patient care services and surgical procedures of a medical residency program have been fully suspended, part of the activities should be relocated to other services. If this is not possible, the decision regarding the continuity of the program must be taken in common agreement between the COREME and local health managers. The same recommendation applies to situations in which medical residents have been called upon to act to face the pandemic in other municipalities.

The SISCNRM also foresees the probability of interrupting the activities of a medical residency program because services are inadequate or for lack of safety for performing professionals in the face of the pandemic. In such cases, the flow should be:

- Record the reasons for interrupting the medical residency program.
- Issue an official information note on the suspension of the medical residency program to residents, preceptors, and managers of the health service.
- Move medical residents to other public or private services so they can complete their training in the specialty or transfer their activities to in-hospital care, assisting in the care of inpatients or management activities.
- Allow the anticipation of the vacation period of the residents.

Having exhausted all the possibilities for maintaining the medical residency program, the situation must be communicated to the State Medical Residency Commission (CEREM, in the Portuguese acronym) and the SISCNRM so that the medical residency program authorization act is reviewed following the consequences provided for in the legislation in force.

## Recommendations

In Brazil, there is a big difference in the situation of the COVID-19 pandemic between states and cities. The great territorial extension and social, cultural, and financial diversity of the country mean that adaptation mechanisms of each program are applied in different degrees and at different times.

Since interruptions related to COVID-19 are likely to continue for many months, the programs will move toward a process of innovation and adaptation that should guarantee residents continuous learning within this new reality.

Considering that the COVID-19 pandemic context represents challenges related to safety concerns, PPE shortages, physical distance, and additional economic and financial responsibilities for hospitals, the SISCNRM of the Brazilian Association of Gynecology and Obstetrics Associations (FEBRASGO, in the Portuguese acronym) proposes this document to guide supervisors, preceptors, and residents. It is aimed at sharing experiences and suggesting resources for medical residency programs while managing the pandemic and maintaining a physical distance.

Based on the available literature and recognizing the nuances of each medical residency program, we divided the main items of recommendations during the COVID-19 pandemic into topics.

## Safety

Safety is an essential item for the development of activities related to a medical residency. The large number of lives lost by health professionals throughout the pandemic in other countries shed even more light on this fundamental item in healthcare and training.^5^ The safety recommendations are:

- Make PPE available to residents and preceptors. If there is a shortage of PPE, the residency coordinator should organize a flow of participation in the procedures. This may require a reduction in the number of residents per procedure.
- Provide training to residents and preceptors on the appropriate use of PPE, including their donning and removal.
- Delay or cancel elective surgeries and nonurgent outpatient clinic visits according to recommendations from the Regional Council of Medicine and specialty societies. Postponing visits and surgeries that may worsen the health status of the patient shall not be recommended.
- Adjust the healthcare provided by residents to minimize exposure and preserve the workforce. Encourage telehealth programs, as suggested by Brazilian legislation. At this point, it is important that the program offers training and mentoring for residents in this type of service.
- Whenever possible, try to increase the number of consultations by telehealth. Schedule in-person consultations only for the selected cases that may harm the health of the woman if there is a postponement, such as oncology, prenatal care, assistance to women victims of sexual violence, sexually transmitted infections, care for women living with HIV and family planning services.^6^ ^7^
–The assistance that requires face-to-face meetings should be performed by a reduced team with the minimum number of residents and preceptors required for the provision of adequate and safe care to patients.
- Medical residents and preceptors included in the risk group according to criteria established by the Ministry of Health must be protected. Whenever possible, avoid contact of these professionals with suspected or confirmed patients for COVID-19. This group can be directed to telehealth visits or other noncare activities. If this is not possible, sick leave may be resorted to.

## Workload and Activities of the Program

In-service medical training, which characterizes the medical residency, will change during the pandemic period. The services will need to adapt their theoretical activities to virtual learning environments. Elective care and procedures will be temporarily compromised.^8^ ^9^

For theoretical activities, the recommendation is to use the maximum workload (total of 12 hours per week) transitioning, as much as possible, from face-to-face courses to distance learning mediated by technologies. Include topics related to individual safety and training of care flows to patients affected by COVID-19 in the theoretical content.^4^

Attention should be paid to the maximum workload in practical activities as established by national guidelines for medical residency programs, which corresponds to 48 hours per week, including shifts.

The workload in obstetrics can be performed in activities such as, prenatal care, childbirth care, postpartum care, and wards of obstetric pathology. The workload in gynecology can be performed in essential activities (gynecological oncology, care for victims of sexual violence, gynecological care, care for sexually transmitted infections, and family planning, among others), according to the possibilities of each program.

The activities performed by residents must be duly registered so they can be counted as part of the workload in the programs.

The form of replacing unfulfilled activities, if needed, will be regulated later by the CNRM.

Given the need to relocate residents for work in activities related to the pandemic, adequate safety, work, and supervision conditions must be ensured.

Maintain a communication channel between supervisor, preceptors, and residents, use virtual meetings to discuss day-to-day problems related to the stress generated by the COVID-19 pandemic and, if possible, provide psychological support to residents and preceptors.

Encourage residents and preceptors to seek online scientific updates such as those promoted by the FEBRASGO and associated institutions.

Guide residents and preceptors regarding the rules established by local ethics committees for conducting clinical research during this period.

## Final Considerations

In consequence of the COVID-19 pandemic, the training of medical residents in gynecology and obstetrics is in a troublesome situation. Aside from the significant concern with the safety, physical, and mental health of preceptors and residents, educational aspects must be considered. The readjustment of programs regarding the minimum number of procedures and scenarios that enable the acquisition of the skills and attitudes provided for in the Competency Framework in Gynecology and Obstetrics^10^ must be performed by each program, respecting the future regulations of the CNRM. At this moment, the innovation and adaptability of those involved will stand out and transform the gynecology and obstetrics training in Brazil. The FEBRASGO National Specialized Commission on Medical Residency reiterates its commitment to assist and guide preceptors and supervisors in the process of training and assessing medical residents during the COVID-19 pandemic.
